# A Single-Centre, Retrospective, Observational Study to Assess Safety and Functional Outcomes of Arthroscopic Meniscal Repair Using Surestitch All Inside Implant

**DOI:** 10.7759/cureus.38221

**Published:** 2023-04-27

**Authors:** Karnav A Panchal, Ashok K Moharana, Sachin Angrish, Deepak TS

**Affiliations:** 1 Arthroscopy and Sports Medicine, Epic Hospital, Gujarat, IND; 2 Clinical Affairs, Healthium Medtech Limited, Bengaluru, IND

**Keywords:** patient-reported outcomes, lysholm score, sane score, tegner level, ikdc score, meniscal tear

## Abstract

Introduction

The meniscus plays a vital role in maintaining knee stability. It acts as a shock absorber and knee filler. The incidence of meniscal tears is estimated to be 60 per 100,000 people. Due to lack of awareness among patients, only 10% of the meniscus tears were treated through partial or total meniscectomy. Recently, the concept of meniscus preservation surgery has emerged to preserve early degeneration of the knee joint. In the current retrospective study, safety and functional outcomes of arthroscopic meniscal repair surgery using Surestitch All inside implants (Sironix Arthroscopy Solutions, Healthium Medtech Limited, Bengalaru, India) were assessed.

Methods

Fifty-two patients who underwent arthroscopic meniscal repair surgery between January 2019 to July 2022 at Epic Hospital in Gujarat, India, were enrolled in the study. Retrospective data including demographics, injury details, surgery details, and post-surgery complications were collected from the medical records of the patients. Then, the patients were followed up telephonically to document safety and functional outcomes using patient-reported instruments such as International Knee Documentation Committee (IKDC) score, Single Assessment Numeric Evaluation (SANE) score, Tegner activity level, and Lysholm knee score.

Results

The recruited patients had the mean age, height, and weight of 37.56 ± 12.52 years, 167.61 ± 7.28 cm, and 75.87 ± 10.7 kgs, respectively. Seventy-one percent of patients were male and 29% were female. Majority of the patients had the routine of doing mild exercise. During pre-surgery representations, medial meniscal tear was observed in majority of patients. The mean length of the tear was 1.32 ± 0.84 cm. In addition, patients were also diagnosed with anterior cruciate ligament (ACL), posterior cruciate ligament (PCL), medial collateral ligament (MCL) tears, and osteochondral defects. Surgeries for meniscal repair were performed using Surestitch All inside implant. In patient-reported outcomes, the mean IKDC, SANE, and Lysholm scores were 81.72 ± 14.23, 94.02 ± 13.79, and 93.32 ± 14.63, respectively. When the mean Tegner scores of pre-injury and post-surgery periods were compared, this resulted in no significant difference (p>0.05) in the activity levels of the patients.

Conclusion

Based on our findings, arthroscopic meniscal repair with Surestitch All inside meniscal repair implant provides satisfactory and favorable functional outcomes with no remarkable adverse events.

## Introduction

The knee is more prone to injury due to its anatomical structure, functional demands, and exposure to external forces. The meniscus plays a vital role in maintaining knee stability as it acts as a shock absorber and knee filler [1]. Menisci transmit about 85-90% of weight-bearing forces during knee flexion and 50% during extension. They compensate for gross abnormality between femoral and tibial articular surfaces and provide chondroprotection through weight distribution by enlarging the contact area between the femur and the tibia [1,2].

An injury to a ligament in the knee often entails damage to other structures because of the complex construction of the joint. The following ligaments enable the knee to support the body’s weight and remain flexible: posterior cruciate ligament (PCL) and anterior cruciate ligament (ACL) control back-and-forth movement; medial collateral ligament (MCL) helps to brace the inside of the knee; and lateral collateral ligament (LCL) braces the outside of the knee, controlling sideways motion and protecting the knee from over-extending [3]. While most injuries to the knee ligaments are sprains or ruptures, sudden impact can result in a partial or complete tear [4].

Notably, meniscal tear and repair is the fastest-growing category. With the increasing interest in sports activities, there is a surge in meniscal injury cases. The incidence of meniscal tears is estimated to be 60 per 100,000 people [3]. However, the true incidence is likely to be grossly underestimated. Evidence in the literature suggests that meniscal injury may lead to an early onset of osteoarthritis [5]. A study by Jarraya et al. found that more than 75% of patients with symptomatic osteoarthritis have a meniscal injury [3]. Due to lack of awareness among patients, only 10% of meniscus tears were repairable in the past. Often, total meniscectomy was the gold standard in the management of meniscal tears [6]. As the meniscus has a weight-bearing function, meniscectomy interferes with the biomechanics of the knee joint, leading to early degenerative changes [7]. Due to these drawbacks, the concept of meniscus preservation surgery has emerged. Meniscal repairs have been fairly successful, with a failure rate of less than 10%. Several techniques such as inside-out technique, meniscal fixators, all-inside technique, and outside-in technique have been developed recently for meniscal repairs [8]. The goals of meniscal repair surgeries are to relieve pain, facilitate preinjury levels for daily living activities, and prevent early degeneration of the knee joint.

To address the gap in patients’ awareness and need for meniscal repairs, Sironix Arthroscopy Solutions (Healthium Medtech Limited, Bengaluru, India) has developed Surestitch^TM^ All inside meniscal repair implant. It is intended for use as a suture retention device to facilitate endoscopic soft tissue fixation. The current retrospective study is aimed to assess the safety and functional outcomes after arthroscopic meniscal repair surgery using Surestitch All inside implant.

## Materials and methods

Study design and ethical approval

A retrospective, observational, single-center, clinical study was conducted at Epic Hospital in Gujarat, India, for patients who underwent arthroscopic meniscal repair surgery between January 2019 to July 2022. After obtaining approval from Epic Hospital Institutional Ethical Committee (IEC), retrospective data were collected from the medical records of the patients. Then, the patients were followed up telephonically in November 2022. All the patients were available over the phone thus, no patient was lost to follow-up or excluded from the study. Verbal informed consent was taken from each participant before their enrolment in the study. The study followed the regulations mentioned in the International Council for Harmonization of Technical Requirements for Pharmaceuticals for Human Use (ICH), the Declaration of Helsinki, Medical Device Rules 2017, and the International Standard Organization (ISO) 14155:2020.

Patients 

Based on inclusion and exclusion criteria, all the patients aged ≥18 years who underwent meniscal repair surgery with Surestitch All inside meniscal repair implant between January 2019 to July 2022, patients who were willing to give informed consent for the study during a telephonic follow-up visit, and patients with associated ACL, MCL, LCL, PCL, and chondral injuries were included in the study. Those patients who have suffered from traumatic injury to the same knee after meniscus repair surgery and could not be contacted after three attempts were excluded.

Objectives

Objectives were: 1. To evaluate the postoperative subjective knee function after meniscal repair using Surestitch implants using International Knee Documentation Committee (IKDC) score and Single Assessment Numeric Evaluation (SANE) score; 2. To review the safety of Surestitch implants in meniscal repair by evaluating adverse events after surgery; 3. To compare the Tegner activity level in patients before the injury and after meniscal repair; and 4. To evaluate the knee-specific symptoms after meniscal repair using the Lysholm knee score.

Surgical procedures

**Figure 1:**
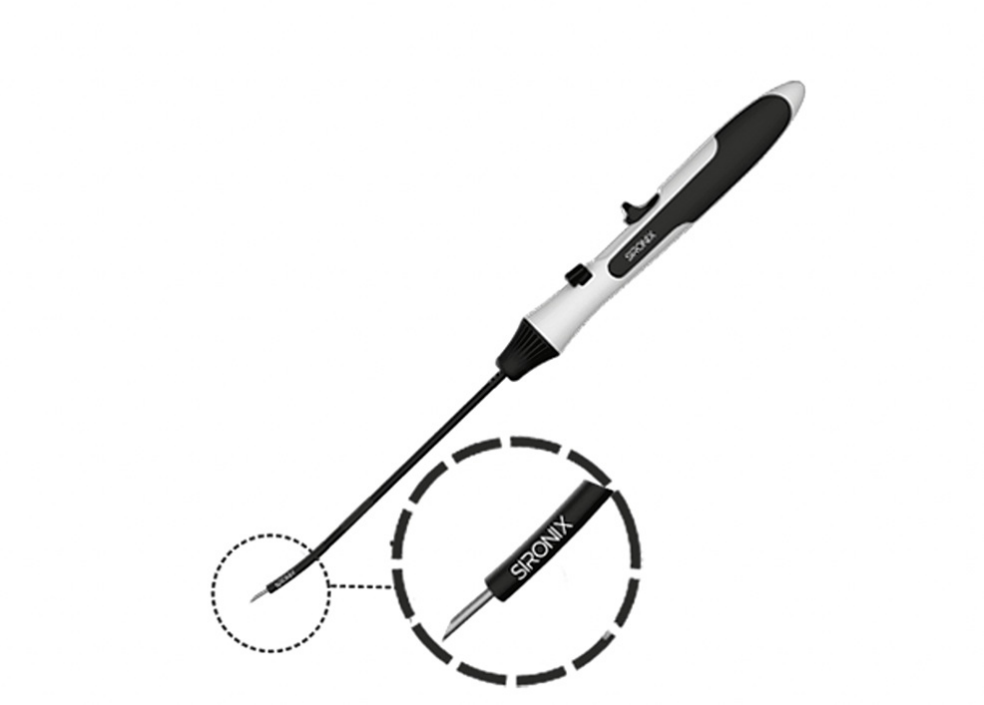
Surestich All inside meniscal repair implant Sironix Arthroscopy Solutions, Healthium Medtech Limited, Bengalaru, India

All the patients were subjected to magnetic resonance imaging (MRI) to review the condition of the injured knee. Based on MRI findings, the meniscal tear was repaired using Surestitch All inside meniscal repair implant and concomitant surgeries were performed by the same surgeon. Depending on the status of the knee injury, the number of implants varied in the patients who underwent meniscal repair surgery. Surestitch implant includes two polyether ether ketone (PEEK) non-absorbable implants, pre-tied with US Pharmacopeia (USP) #2-0 non-absorbable ultra high molecular weight polyethylene (UHMWPE) suture and preloaded into a needle delivery system (Figure 1). Briefly, the reparability and anteroposterior length of the meniscus was assessed using a graduated probe. Depending on the meniscus thickness, the needle's depth was adjusted by rotating the depth control knob. The Surestitch was slid up to the meniscus in the inverted position with the help of cannula. When the depth control tube touched the meniscus, the safety knob was turned to active mode followed by deployment of implants using deployment knob. The needle was retracted carefully and the suture tail was pulled using a knot pusher and cut accordingly. In addition, ACL reconstruction was also performed as an associated surgery in a few patients.

Data collection

The retrospective data were collected from medical records of the patients which include: baseline characteristics such as sex, age, height, weight, marital status, and exercise participation; injury details: location, mechanism of injury, rupture type, and type of tear; surgery details: no of implants used, associated surgeries, complications, and re-operations. Thereafter, all the patients were contacted and followed up telephonically to obtain functional outcomes of meniscal repair surgery by assessing IKDC subjective evaluation, Lysholm knee score, SANE score, and Tegner activity score. Data of IKDC, Lysholm knee score, and Tegner activity levels were collected, as described previously [9].

SANE

SANE is a simple method of evaluating patients' sense of functional improvement after meniscal repair surgery and rating for current illness score about their pre-injury baseline on a range of 0 to 100. SANE scores are most commonly used by orthopedic sports specialist surgeons, and usually for the shoulder and the knee [10]. 

Statistical analysis

Statistical analyses were conducted using GraphPad software v8.0. Descriptive data were generated and reported in mean ± standard deviation, range, or proportions. The mean Tegner levels before the injury and after surgery were compared using a t-test. A significance level of ≤ 0.05 was considered. 

## Results

Participants 

Fifty-two patients met the inclusion criteria and were included in the study. These patients underwent meniscal repair surgery between January 2019 to July 2022. All were followed up telephonically in November 2022. The recruited patients had a mean age of 37.56 ± 12.52 years (range: 18-52 years). In the study, 71% of patients were male (n=37/52) and 29% were female (n=15/52). The mean height and weight of the recruited patients were 167.61 ± 7.28 cm and 75.87 ± 10.7 kgs, respectively. All the recruited participants were Indian belonging to different states of India, viz. Gujarat (n=45), Rajasthan (n=6), and Madhya Pradesh (n=1). Out of 52 participants, 39 (75%) were married, 12 (23%) were single, and one (2%) was a divorcee. Referring to employment status, 25 patients (48%) were businessmen or full-time working employees (>8 hours) and 27 (52%) were non-workers. Among the non-working patients, 14 patients (27%) were housewives, nine (17%) were students, and the remaining four (8%) had retired from their work. When participants were asked about their exercise routine, 73% (n=38/52) did “mild” exercise whereas 27% were not involved in any kind of exercise. None of the patients had an exercise routine of moderate or severe types. The demographic characteristics of the patients are listed in Table 1.

**Table 1:** Demographic characteristics of the patients n=number of patients, %=percentage, SD=standard deviation

Characteristics, n (%)	Number of patients (n= 52)
Age (years; Mean ± SD)	37.56 ± 12.52
Gender	
Male	37 (71)
Female	15 (29)
Height (cm; Mean ± SD)	167.61 ± 7.28
Weight (kg; Mean ± SD)	75.87 ± 10.7
Ethnicity: Asian	52 (100)
Marital status	
Single	12 (23)
Married	39 (75)
Divorcee	01 (02)
Employment status	
Full working (>8 hours)	25 (48)
Not working	27 (52)
Occupation	
Business	13 (25)
Employee	12 (23)
Household	14 (27)
Student	09 (17)
Retired employees	04 (8)
Exercise	
No	38 (73)
Mild	14 (27)

Details of the injury

A slight predominance was observed on the right side of the knee i.e., 56% of patients (n=29) had right knee injury vs. 44% (n=23) with left knee injury. The mechanism of injury included falls while performing daily activities or sports or an accident. About 56% of patients (n=29/52) got injuries during daily activities, 19 (37%) while participating in recreational sports, and four patients met with an accident. After the injury, 65% of patients (n=34) had “moderate” pain, 18% (n=10) had “severe” pain, and the remaining eight (17%) reported “mild” pain as a symptom. The mean time period of injury to the date of surgery was 3.39 ± 3.22 months. The details of the injuries are listed in Table 2.

**Table 2:** Details of the injury n=number of patients, %=percentage, SD=standard deviation

Characteristics, n (%)	Number of patients (n= 52)
Knee injury	
Right	29 (56)
Left	23 (44)
Mechanism of injury	
Daily activities	29 (56)
Sports	19 (37)
Accidents	04 (07)
Pain	
Mild	8 (17)
Moderate	34 (65)
Severe	10 (18)
Time of surgery since injury (months; Mean ± SD)	3.39 ± 3.22

MRI findings

Medial meniscal tear was more prevalent (75%; n=39) than lateral tear (21%; n=11). Only two patients (4%) had both medial and lateral tears. On defining the orientation of meniscal tears, 46% of patients (n=24) had vertical tears followed by radial tears in 16 patients (32%). The horizontal and buckle handle type of tears were observed in five (10%) and six (12%) patients, respectively. The mean length of the tear was calculated to be 1.32 ± 0.84 cm. About 65% of patients (n=34/52) had a red-red zone of rupture whereas the remaining 35% (n= 18/52) had a red-white zone of rupture. In MRI reports, patients were diagnosed with ACL tear, PCL tear, MCL tear, osteochondral defects involving femoral condyle, and loose body within the joint other than meniscal tear. 

Surgeries for meniscal repair were carried out using Surestitch All inside implant. Two implants were used in about 46% of patients (n=24), one implant in 44% (n=23), and three implants in 6% (n=3). Four and five implants were used in one patient each. The mean number of days hospitalized was estimated as 2.16 ± 0.65 days. The details of the surgery are shown in Table 3.

**Table 3:** Details of the surgery MRI=magnetic resonance imaging, ACL=anterior cruciate ligament, PCL=posterior cruciate ligament, MCL=medial collateral ligament, N=number of patients, %=percentage, SD=standard deviation

Characteristics, n (%)	Number of patients (n= 52)
MRI findings	
Meniscal tear	52 (100)
ACL tear	41 (79)
ACL+PCL+MCL	02 (04)
Osteochondral defects	02 (04)
Loose body within the joint	02 (04)
Meniscal tear	
Medial	39 (75)
Lateral	11 (21)
Both	02 (04)
Type of meniscal tear	
Horizontal	05 (10)
Vertical	24 (46)
Radial	16 (32)
Buckle handle	06 (12)
Length of tear (cm, Mean ± SD)	1.32 ± 0.84
Zone of rupture	
Red-red	34 (65)
Red-white	18 (35)
Number of days hospitalized (days, Mean ± SD)	2.16 ± 0.65
Number of implants	
1	23 (44)
2	24 (46)
3	03 (06)
4	01 (02)
5	01 (02)

Patient-reported outcomes

Adverse Events

The patients were followed up for a mean time of 16.35 ± 9.23 months. During the study or follow-up period, no patient reported any kind of adverse event, re-injury, or any other postoperative complications. 

Subjective Knee Function and Knee-Specific Symptoms

Regarding subjective knee function, the mean IKDC score was calculated and found to be 81.72 ± 14.23. About 48% of patients (n=25) had an above 90 IKDC score, 25% (n=13) had a score in the range of 80-89, and the remaining 27% (n= 14) had a score below 80. Further, the mean SANE score was found to be 94.02 with a standard deviation of 13.79.

**Figure 2:**
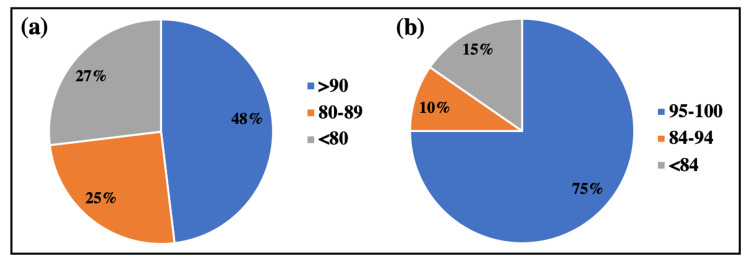
Patient-reported outcomes after meniscal repair. Pie charts representing the percentage of patients showing functional outcomes using (a) IKDC score and (b) Lysholm score. IKDC=International Knee Documentation Committee

For knee-specific symptoms, the postoperative mean score of the patients was found to be 93.32 ± 14.63. About 39 patients (75%) had excellent scoring (range: 95-100), suggesting no knee symptom, five patients had good scoring (range: 84-94), and 15% (n=8) had fair range (<84). The proportion of patients in different IKDC and Lysholm ranges is shown in Figure 2.

Tegner Knee Activity

**Figure 3:**
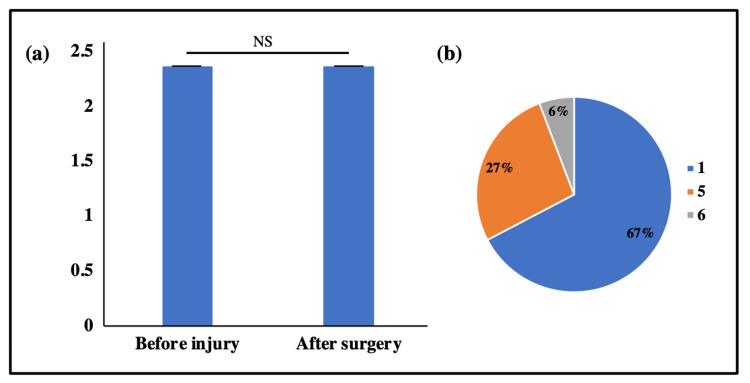
Tegner activity level. (a) Bar graphs representing mean Tegner activity before the injury and after surgery and (b) Pie-chart representing the proportion of patients with Tegner activity levels of 1, 5, and 6. NS=non-significant

The Tegner scale was used to assess and compare the activity level of the patients for pre-injury and post-surgery periods. On comparing the mean Tegner scores, no significant difference was observed between pre-injury and post-surgery levels (2.34 ± 1.23 vs. 2.34 ± 1.23, p>0.05), suggesting the patients had attained similar Tegner levels of pre-injury period in the postoperative period. Majority of the patients (67%, n=35/52) had a Tegner activity level of 1 and the remaining had 5 (14/52) or 6 (3/52). The data are shown in Figure 3.

## Discussion

The present study comprised 52 patients who underwent meniscal repair surgery and were assessed for functional outcomes using IKDC, Tegner, Lysholm, and SANE scales. All the patients were operated on using Surestitch All inside meniscal repair implants. A total of 52 patients who underwent meniscal repair surgery between January 2019 to July 2022 were recruited in the study based on inclusion and exclusion criteria. In our study, all the recruited patients were aged between 18 to 52 years with a mean age of 37.56 ± 12.52 years. In a study, Ventura et al. (2023) suggested that age should not be considered a contraindication for meniscal repair surgeries [11]. The male preponderance of 71% (n=37/52) was observed while 29% of patients (n=15/52) were female. In 2022, Robinson et al. conducted a retrospective cohort study of 88 patients who underwent meniscal repair along with ACL reconstruction in which 74% were males and 26% were females [12]. The gender of recruited patients is similar to our study recruitments. The mean height and weight of the recruited patients were 167.61 ± 7.28 cm and 75.87 ± 10.7 kgs, respectively. All the recruited participants were Asians. Substantial evidence in the literature suggests that the time since the injury to surgery can range from days to weeks and years as there is no ideal time [13,14]. 

Based on the spatial orientation and pattern, meniscal injuries are categorized as medial and lateral, and vertical, horizontal, bucket-handle, and radial type. These tears are accompanied by osteoarthritic changes. In our study, medial meniscal tear was more prevalent (75%) than lateral tear (21%). In two individual studies conducted by Buchbinder et al. (2016) [15] and Ventura et al. (2023) [11], the authors observed medial meniscus tears in the majority of the patients. The findings of these two studies favor our data. On defining the type of meniscal tear, 46% of patients had vertical tear followed by radial tear in 16 patients (32%). The horizontal and buckle handle type of tears were observed in five (10%) and six (12%) patients, respectively. 

Thereafter, the patients were contacted by telephone, and data on patient-reported outcomes in terms of IKDC, Lysholm, Tegner activity, and SANE scales were documented. Primarily, the subjective knee function was assessed postoperatively using the IKDC questionnaire and the mean score was 81.72 ± 14.23. In the study of Robinson et al. (2022) [12], the IKDC score of patients was reported as 82.8, which is similar to our study. The findings of our study are in concordance with the study of Robinson et al. (2022). Further, the mean SANE score was found to be 94.02 ± 13.79 in our study. A cohort study was conducted by Bailey et al. (2021) [16] to determine the effects of intraoperative platelet-rich plasma (PRP) on postoperative knee function and complications at two years after ACL reconstruction with meniscal repair. In this study, the authors observed SANE knee function scores as 91.6 ± 11.2 vs 92.4 ± 10.6 between PRP and control group, which are similar to our findings. 

Thereafter, the postoperative knee-specific symptoms were evaluated using Lysholm score. The postoperative mean score of the patients was found to be 93.32 with a standard deviation of 14.63. In a study, Abdallah et al. (2020) [17] evaluated clinical and radiological outcomes following meniscal repair using different arthroscopic techniques in 61 patients. The authors observed the mean Lysholm score of 93.26 ± 2.95 at 12-month follow-up. The data are in accordance with our findings. 

Limitations

There are a few limitations of this study. This was a retrospective study with a small sample size. Therefore, the success of the Surestitch implant could not be compared with any other implant or with a control group. Only patient-reported outcome measures were taken into consideration without any physical verification.

## Conclusions

The meniscus is an important shock absorber present between the knee joint. Meniscal tear may lead to osteoarthritis of the knee joint. Lysholm, IKDC, SANE scores, and Tegner activity levels are considered useful patient-reported instruments to evaluate the functional outcomes after surgery. Based on our findings of IKDC, SANE, Lysholm scorings, and Tegner activity levels, arthroscopic meniscal repair with Surestitch All inside meniscal repair implant provides satisfactory and favorable functional outcomes with no remarkable adverse events.
